# Rural-Urban Differences in Breast Cancer Surgical Delays in Medicare Beneficiaries

**DOI:** 10.1245/s10434-022-11834-4

**Published:** 2022-05-24

**Authors:** Ronnie J. Zipkin, Andrew Schaefer, Changzhen Wang, Andrew P. Loehrer, Nirav S. Kapadia, Gabriel A. Brooks, Tracy Onega, Fahui Wang, Alistair J. O’Malley, Erika L. Moen

**Affiliations:** 1 Department of Biomedical Data Science, Geisel School of Medicine at Dartmouth, Lebanon, NH USA; 2 The Dartmouth Institute for Health Policy and Clinical Practice, Lebanon, NH USA; 3 Department of Geography and Anthropology, Louisiana State University, Baton Rouge, LA USA; 4 Department of Surgery, Geisel School of Medicine at Dartmouth, Lebanon, NH USA; 5 Dartmouth Cancer Center, Dartmouth-Hitchcock Medical Center, Lebanon, NH USA; 6 Department of Medicine, Geisel School of Medicine at Dartmouth, Lebanon, NH USA; 7 Huntsman Cancer Institute, University of Utah, Salt Lake City, UT USA; 8 Department of Population Sciences, University of Utah, Salt Lake City, UT USA; 9 Department of Epidemiology, Geisel School of Medicine at Dartmouth, Lebanon, NH USA

## Abstract

**Background:**

Delays between breast cancer diagnosis and surgery are associated with worsened survival. Delays are more common in urban-residing patients, although factors specific to surgical delays among rural and urban patients are not well understood.

**Methods:**

We used a 100% sample of fee-for-service Medicare claims during 2007–2014 to identify 238,491 women diagnosed with early-stage breast cancer undergoing initial surgery and assessed whether they experienced biopsy-to-surgery intervals > 90 days. We employed multilevel regression to identify associations between delays and patient, regional, and surgeon characteristics, both in combined analyses and stratified by rurality of patient residence.

**Results:**

Delays were more prevalent among urban patients (2.5%) than rural patients (1.9%). Rural patients with medium- or high-volume surgeons had lower odds of delay than patients with low-volume surgeons (odds ratio [OR] = 0.71, 95% confidence interval [CI] = 0.58–0.88; OR = 0.74, 95% CI = 0.61–0.90). Rural patients whose surgeon operated at ≥ 3 hospitals were more likely to experience delays (OR = 1.29, 95% CI = 1.01–1.64, Ref: 1 hospital). Patient driving times ≥ 1 h were associated with delays among urban patients only. Age, black race, Hispanic ethnicity, multimorbidity, and academic/specialty hospital status were associated with delays.

**Conclusions:**

Sociodemographic, geographic, surgeon, and facility factors have distinct associations with > 90-day delays to initial breast cancer surgery. Interventions to improve timeliness of breast cancer surgery may have disparate impacts on vulnerable populations by rural-urban status.

**Supplementary Information:**

The online version contains supplementary material available at 10.1245/s10434-022-11834-4.

For the approximately one in eight women in America who will develop breast cancer in their lifetime, early diagnosis and timely receipt of treatments, such as surgery, chemotherapy, or radiation, represent important measures of care quality.^1,2^ In addition to being associated with increased burdens of patient stressors, delayed care after diagnosis has been linked to inferior survival.^3–6^ Many contributors to the duration of the preoperative period of recently diagnosed patients are clinically appropriate and include surgical scheduling logistics requisite to case complexity, transfers of care, the seeking of second opinions, imaging, and treatment with neoadjuvant chemotherapy.^7–10^ For patients with early-stage disease, however, surgical delays beyond 60 days have been associated with tumor and nodal upstaging,^11,12^ as well as decreased survival,^4,12–15^ with longer delays having a more pronounced adverse impact. Whereas numerous guidelines and quality measures have been established to define clinically appropriate intervals between breast cancer diagnosis and initiation of adjuvant chemotherapy, radiation, or endocrine therapy, guidelines specifying a recommended interval from diagnosis to surgery were lacking until 2020.^16,17^ After elective surgical procedures in the United States were subjected to significant delays following the onset of the coronavirus disease 2019 (COVID-19) pandemic, the COVID-19 Pandemic Breast Cancer Consortium convened in early 2020 and specified in their updated triage and management recommendations that surgical delays more than 90 days may adversely affect outcomes for many breast cancer patients.

Among patient-level variables consistently associated with surgical delays are increased age at diagnosis, non-Hispanic (NH)-black or Hispanic race/ethnicity, multimorbidity, and urban residence.^9,12–14,18–22^ Drivers of delay specific to urban patients remain little understood, nor it is clear why rural patients experience fewer surgical delays, considering they are more likely to encounter barriers to care, such as scarcity of specialists or nearby facilities for mammography, imaging, or treatment.^21,22^ Although 20% of Americans live in rural areas, nationwide studies of cancer care are frequently limited to data from the Surveillance, Epidemiology, and End Results (SEER)-Medicare registry and National Cancer Database (NCDB). Until the formation of SEER 21 in 2018, SEER registries represented regions encompassing under 30% of Americans, among which rural areas were substantially underrepresented.^23^ The NCDB registry covers only a third of hospitals in the United States and may undersample hospitals with rural catchment areas.^24^ Additionally, few studies of breast cancer surgical delay have taken surgeon attributes into account.^10^ In this study, we use nationwide Medicare claims data to identify patient, surgeon, and facility characteristics associated with surgical delays specific to rural and urban patients.

## Methods

### Medicare Beneficiary Data and Claims

Our dataset included the 100% sample of fee-for-service Medicare beneficiaries over 2007–2014, prior to the Centers for Medicare and Medicaid Services’ (CMS) 2015 adoption of the *International Classification of Diseases, 10th edition, Clinical Modification* coding paradigm. Claims and characteristics associated with patients, their treating physicians, and facilities where clinical encounters occurred were identified from the CMS Master Beneficiary Summary, Medicare Provider Analysis and Review, Carrier, and Outpatient services files. All study protocols were approved by the institutional review board at Dartmouth College.

#### Study Cohort

Women with incident breast cancer diagnoses were identified by a modification of the biopsy and surgery algorithms of Bronson et al. (Supplemental Table 1), selecting for the earliest diagnosis date to exclude cases of disease recurrence.^25^ Patients were included if they received definitive surgical treatment with therapeutic intent 1–180 days after their first needle biopsy (Supplemental Table 2).^13,19^ Women who received neoadjuvant chemotherapy were excluded, as longer biopsy-to-surgery intervals would be clinically appropriate.^10,20^ Patients with claims indicating distant metastatic disease were excluded. Lastly, patients were excluded who had missing ZIP codes for residence or surgery facility or if they did not have an identifiable surgeon.

### Outcomes

The primary outcome was substantial surgical delay, defined as definitive surgery performed > 90 days after a patient’s initial needle biopsy, which we treated as a binary outcome, given that such a window consistently demonstrates negative impacts on survival.^4,12–14^ Because surgery > 60 days after diagnosis has been reported as a potential threshold for worsened survival in data from multiple registries,^12,13^ we performed secondary analyses for biopsy-to-surgery intervals > 60 days.

### Independent Variables

Patient rurality was first assessed as an independent variable, then used to stratify the patient cohort by rural or urban ZIP codes of residence. ZIP code-level rurality was assigned using secondary Rural-Urban Commuting Area (RUCA) designations from the 2010 RUCA to ZIP code file based on the patient’s ZIP code of residence in the year of diagnosis and the facility where surgery was performed.^26^ We aggregated RUCA codes using University of Washington Categorization A.^27^ Area deprivation index (ADI) by ZIP code, reflecting a score from least (ADI = 1) to most socioeconomically disadvantaged (ADI = 100) was obtained from the University of Wisconsin Neighborhood Atlas and assigned by the patient’s 9-digit ZIP code of residence.^28^ For ZIP codes without a ZIP+4 ADI rank, the mode of ZIP+4 ADIs in a 5-digit ZIP code was used. The mode of ZIP+4 ADIs in the parent ZIP code tabulation area (ZCTA) was used if this was unavailable.

To estimate travel times from patient residence to the facility where they received surgery, ZIP code-to-ZIP code origin-destination (OD) pair driving times were obtained from the national drive time matrix computed by Hu and colleagues,^29^ which accounted for the hierarchical structure of road networks and real-time traffic for OD pairs and intrazonal trips (Supplemental Appendix 1).

Additional patient-level covariates included age, race/ethnicity, the presence of comorbidities, the number of clinical encounters taking place between the biopsy and surgery, and census region. Charlson comorbidity conditions were identified for patients by the method of Klabunde from claims over the 12 months preceding the diagnosis date.^30^

Hospital-level covariates included teaching hospital and NCI-designated cancer center status, as identified in American Hospital Association data, and rurality.

Physician-level covariates included gender, patient volume, and the number of hospitals at which the surgeon operated. Gender was identified using the National Plan and Provider Enumeration System National Downloadable File.^31^ The number of patients for whom breast cancer-directed surgery was performed, and of these the number of hospitals at which the surgeon operated were identified by year. Surgeon volume among cohort patients in the year of the patient’s diagnosis—irrespective of surgery type—was categorized as low (< 5 cases), medium (5–9 cases), or high (> 9 cases), defined based on prior work by Nattinger and colleagues on the surgeon-volume relationship that was similarly limited to counts of female Medicare beneficiaries with early-stage breast cancer.^32^

### Statistical Analysis

Patient characteristics were summarized with descriptive statistics by extent of delay. Because surgery with immediate reconstruction entails additional planning and scheduling constraints and is associated with a 14-day increase in median biopsy-to-surgery times,^18,19,33^ a separate analysis was performed for patients whose operations included immediate reconstruction. Comparisons between groups were performed using the Kruskal-Wallis test for quantitative variables and chi-squared tests for categorical variables. To identify characteristics associated with clinically significant surgical delays, mixed effects logistic regression was used to model each delay type as a binary outcome, with random effects for operating surgeon and patient county of residence. We then examined associations between delay and travel time, facility rurality, and surgeon characteristics when stratifying patients by rurality of residence—dichotomizing RUCA categories into urban-focused and nonurban-focused (“rural”)—controlling for patient age, race/ethnicity, and the presence of comorbid illness.

## Results

**Fig. 1:**
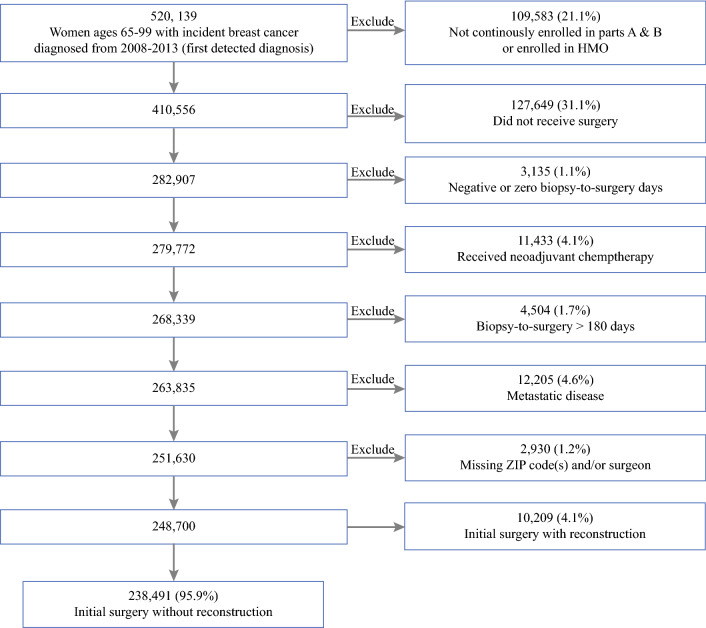
Sample selection

After exclusions, we identified 248,700 women from the overall Medicare fee-for-service population with incident diagnoses of breast cancer made in 2008–2013, of which a final cohort of 238,491 had surgical resection without immediate reconstruction (Fig. 1). The median age was 75 years (interquartile range [IQR] 70–80). Comprising the cohort, 89.8% of women were NH-white and 7.1% were NH-black. The median biopsy-to-surgery interval was 26 days (IQR 17–37). In total, 7.4% of patients experienced intervals > 60 days and 2.4% of patients had delays > 90 days (Table 1). Patients experiencing substantial delays were older, were more likely to be Hispanic or NH-black, had more comorbid illnesses, and were more likely to have received surgery at a teaching hospital or NCI-designated cancer center. Of women undergoing surgical treatment with immediate reconstruction, 1.9% of rural patients and 2.5% of urban patients had surgical delays > 90 days.

**Table 1:** Patient characteristics by surgical delay status for nonreconstruction surgery

	≤ 60 days	> 60 and ≤ 90 days	> 90 and ≤ 180 days	*P*^a^
N	220,892	11,931	5668	
Age (%)				< 0.001
66–69	45,411 (20.6)	2214 (18.6)	1030 (18.2)	
70–74	61,021 (27.6)	3194 (26.8)	1354 (23.9)	
75–79	51,681 (23.4)	2768 (23.2)	1302 (23.0)	
80–99	62,779 (28.4)	3755 (31.5)	1982 (35.0)	
Race/ethnicity (%)				< 0.001
Asian/Pacific Islander	2115 (1.0)	150 (1.3)	68 (1.2)	
Hispanic	1441 (0.7)	174 (1.5)	86 (1.5)	
NH-black	14,588 (6.6)	1494 (12.5)	826 (14.6)	
NH-white	199,680 (90.4)	9866 (82.7)	4572 (80.7)	
Other^b^	3068 (1.4)	247 (2.1)	116 (2.0)	
Charlson comorbidities (%)				< 0.001
0	121,946 (55.2)	5557 (46.6)	2461 (43.4)	
1	59,532 (27.0)	3315 (27.8)	1554 (27.4)	
2 or more	39,414 (17.8)	3059 (25.6)	1653 (29.2)	
Preoperative clinician visits (median [IQR])	0 [0,1]	1 [0,2]	1 [0,2]	< 0.001
Teaching hospital (%)	38,776 (17.6)	3119 (26.1)	1419 (25.0)	< 0.001
NCI cancer center (%)	13,091 (5.9)	1495 (12.5)	667 (11.8)	< 0.001
Est. driving time to hospital				< 0.001
≤ 29 min	84,765 (38.4)	4666 (39.1)	2208 (39.0)	
30–59 min	84,365 (38.2)	4397 (36.9)	2115 (37.3)	
60–179 min	33,345 (15.1)	1734 (14.5)	839 (14.8)	
≥ 180 min	18,417 (8.3)	1134 (9.5)	506 (8.9)	
Region (%)				< 0.001
Midwest	54,656 (24.7)	2300 (19.3)	1136 (20.0)	
Northeast	41,432 (18.8)	3099 (26.0)	1354 (23.9)	
South	86,999 (39.4)	3971 (33.3)	2092 (36.9)	
West	37,805 (17.1)	2561 (21.5)	1086 (19.2)	
Area deprivation index				
Median [IQR]	44 [22,67]	40 [18,66]	42 [21,68]	< 0.001
Missing	730 (0.3)	41 (0.3)	20 (0.4)	
Patient RUCA (%)				< 0.001
Isolated small rural town	8816 (4.0)	357 (3.0)	191 (3.4)	
Small rural town	12,167 (5.5)	452 (3.8)	232 (4.1)	
Large rural city/town	22,787 (10.3)	901 (7.6)	479 (8.5)	
Urban	177,122 (80.2)	10,221 (85.7)	4766 (84.1)	
Surgery RUCA (%)				< 0.001
Isolated small rural town	1218 (0.6)	41 (0.3)	25 (0.4)	
Small rural town	4492 (2.0)	135 (1.1)	75 (1.3)	
Large rural city/town	20,488 (9.3)	658 (5.5)	351 (6.2)	
Urban	194,694 (88.1)	11,097 (93.0)	5217 (92.0)	

*IQR* Interquartile range, *NCI* National Cancer Institute, *NH* Non-hispanic, *RUCA* Rural-urban commuting area

^a^Two-sided Chi-squared test *P* values for categorical variables, Kruskal-Wallis test *P* values for medians

^b^“Other” aggregates patients whose race/ethnicity is identified by the Research Triangle Institute algorithm as: American Indian/Alaskan Native, Other, or Unknown

Multivariable analysis (Table 2) showed an association between > 90-day delays and increased age (older than 80 years: odds ratio [OR] = 1.64, 95% confidence interval [CI] = 1.51–1.78). Compared with NH-white patients, NH-black, Hispanic, and others had higher odds of > 90-day delays (respectively: OR = 1.98, 95% CI = 1.81–2.16; OR = 1.69, 95% CI = 1.33–2.15; OR = 1.43, 95% CI = 1.17–1.75). Longer estimated driving times were associated with higher odds of delays, and patients in the highest ADI quartile had 20% increased odds of delay. Patients operated on by female surgeons had 18% increased odds of experiencing delays. Those operated on by a medium- or high-volume surgeon had decreased odds of delay. Patients having additional clinical encounters between their biopsy and initial surgery were considerably more likely to experience delays. Results from the secondary analysis of > 60-day delay are reported in Supplemental Table 3.

**Table 2:** Models of nonreconstruction surgical delays > 90 days

*N* = 238,491	Crude OR (95% CI)	*P*	Adjusted OR (95% CI)	*P*
*Patient characteristics*				
Age (Ref: 66–69)				
70–74	0.97 (0.89,1.06)	0.51	0.99 (0.91,1.08)	0.85
75–79	1.11 (1.02,1.21)	0.01	1.18 (1.08,1.29)	< 0.001
80–99	1.40 (1.29,1.51)	< 0.001	1.64 (1.51,1.78)	< 0.001
Race/ethnicity (Ref: NH-white)				
Asian/Pacific Islander	1.13 (0.87,1.46)	0.35	1.13 (0.88,1.47)	0.33
Hispanic	1.88 (1.48,2.37)	< 0.001	1.69 (1.33,2.15)	< 0.001
NH-black	2.17 (2.00,2.36)	< 0.001	1.98 (1.81,2.16)	< 0.001
Other^a^	1.44 (1.18,1.75)	< 0.001	1.43 (1.17,1.75)	< 0.001
Charlson comorbidities (Ref: 0)				
1	1.28 (1.19,1.36)	< 0.001	1.25 (1.17,1.34)	< 0.001
2 or more	1.99 (1.87,2.13)	< 0.001	1.95 (1.82,2.09)	< 0.001
Preoperative clinician visits (Ref: 0)				
1	1.31 (1.22,1.42)	< 0.001	1.45 (1.35,1.56)	< 0.001
2 or more	4.20 (3.94,4.48)	< 0.001	4.64 (4.34,4.96)	< 0.001
Treated at teaching hospital	1.42 (1.31,1.54)	< 0.001	1.20 (1.10,1.30)	< 0.001
Treated at NCI cancer center	1.85 (1.65,2.08)	< 0.001	1.41 (1.24,1.59)	< 0.001
Estimated driving time to hospital (Ref: ≤ 29 min)				
30–59 min	1.01 (0.95,1.08)	0.79	1.04 (0.97,1.11)	0.267
60–179 min	1.09 (1.00,1.20)	0.04	1.12 (1.02,1.24)	0.017
≥ 180 min	1.13 (1.02,1.26)	0.02	1.10 (0.99,1.23)	0.077
*Surgeon characteristics*				
Female gender (Ref: Male)	1.28 (1.19,1.37)	< 0.001	1.18 (1.09,1.26)	< 0.001
Patient volume (Ref: Low)				
Medium	0.90 (0.81,0.99)	0.02	0.88 (0.80,0.97)	0.01
High	0.94 (0.86,1.02)	0.12	0.80 (0.73,0.88)	< 0.001
No. hospital affiliations^b^ (Ref: 1)				
2	1.05 (0.98,1.13)	0.15	1.08 (1.01,1.15)	0.03
3 or more	1.03 (0.93,1.14)	0.59	1.03 (0.93,1.14)	0.54
*Regional characteristics*				
Region (Ref: South)				
Midwest	0.78 (0.70,0.86)	< 0.001	0.86 (0.78,0.95)	0.003
Northeast	1.31 (1.17,1.47)	< 0.001	1.25 (1.12,1.38)	< 0.001
West	1.08 (0.96,1.22)	0.19	1.16 (1.04,1.30)	0.007
Area deprivation index (Ref: Quartile 1)				
Missing	1.19 (0.75,1.89)	0.46	1.20 (0.75,1.91)	0.44
Quartile 2	1.09 (1.01,1.18)	0.03	1.11 (1.03,1.20)	0.007
Quartile 3	1.06 (0.97,1.15)	0.19	1.09 (1.00,1.19)	0.05
Quartile 4	1.27 (1.16,1.39)	< 0.001	1.20 (1.09,1.32)	< 0.001
Patient RUCA (Ref: Small rural town)				
Isolated small rural town	1.14 (0.93,1.39)	0.20	1.15 (0.93,1.40)	0.19
Large rural city/town	1.09 (0.92,1.29)	0.30	1.14 (0.96,1.36)	0.13
Urban	1.20 (1.04,1.39)	0.01	1.09 (0.93,1.28)	0.29
Surgery RUCA (Ref: Small rural town)				
Isolated small rural town	1.20 (0.73,1.98)	0.46	1.01 (0.61,1.67)	0.97
Large rural city/town	1.00 (0.76,1.30)	0.98	0.92 (0.70,1.22)	0.55
Urban	1.37 (1.07,1.75)	0.01	1.10 (0.85,1.42)	0.47

Mixed-effects logistic regression of surgical delay greater than 90 days. Random effects for surgeon and patient county of residence

*CI* Confidence interval, *NCI* National Cancer Institute, *NH* Non-hispanic, *OR* Odds ratio, *RUCA* Rural-urban commuting area

^a^“Other” aggregates patients whose race/ethnicity is identified by the Research Triangle Institute algorithm as: American Indian/Alaskan Native, Other, or Unknown

^b^Number of unique hospitals at which a surgeon operated on an early-stage breast cancer patient irrespective of reconstruction status in the year of the patient’s surgery

When patients were stratified by rurality, we were able to identify factors associated with > 90-day surgical delay that were common across the overall cohort as well as factors unique to rural or urban patients (Table 3). Distinct to rural patients, being operated on by a medium-volume or high-volume surgeon was associated with a substantially lower odds of delay (respectively: OR = 0.71, 95% CI = 0.58–0.88; OR = 0.74, 95% CI = 0.61–0.90). Rural patients whose surgeon operated at ≥ 3 hospitals were more likely to experience a delay (OR = 1.29, 95% CI = 1.01–1.64, Ref: 1 hospital). Distinct to urban patients, being treated at a teaching hospital, greater travel time to surgery center, and residing in the Northeast or West increased the likelihood of > 90-day surgical delay.

**Table 3:** Model of nonreconstruction surgical delay greater than 90 days, stratified by rurality

	Rural (*N* = 46,382)		Urban (*N* = 192,109)	
	Adjusted OR (95% CI)	*P*	Adjusted OR (95% CI)	*P*
*Patient characteristics*				
Age (Ref: 66–69)				
70–74	0.94 (0.77,1.15)	0.55	1.00 (0.91,1.10)	0.96
75–79	1.02 (0.83,1.26)	0.84	1.21 (1.10,1.33)	< 0.001
80–99	1.51 (1.24,1.84)	< 0.001	1.66 (1.52,1.82)	< 0.001
Race/ethnicity (Ref: NH-white)				
Asian/Pacific Islander	0.49 (0.06,3.78)	0.49	1.15 (0.89,1.50)	0.28
Hispanic	0.99 (0.35,2.83)	0.98	1.76 (1.38,2.25)	< 0.001
NH-black	1.68 (1.26,2.24)	< 0.001	2.01 (1.83,2.21)	< 0.001
Other^a^	1.87 (1.20,2.91)	0.005	1.34 (1.07,1.67)	0.009
Charlson comorbidities (Ref: 0)				
1	1.31 (1.11,1.55)	0.001	1.24 (1.15,1.33)	< 0.001
2 or more	2.16 (1.82,2.56)	< 0.001	1.91 (1.77,2.06)	< 0.001
Preoperative clinician visits (Ref: 0)				
1	1.62 (1.34,1.96)	< 0.001	1.42 (1.30,1.53)	< 0.001
2 or more	6.03 (5.12,7.11)	< 0.001	4.41 (4.10,4.74)	< 0.001
Treated at teaching hospital	1.22 (0.96,1.55)	0.11	1.19 (1.09,1.30)	< 0.001
Treated at NCI cancer center	1.50 (1.11,2.03)	0.007	1.40 (1.23,1.59)	< 0.001
Est. driving time to hospital (Ref: ≤ 29 min)				
30–59 min	1.13 (0.89,1.43)	0.32	1.03 (0.97,1.11)	0.33
60–179 min	1.11 (0.87,1.42)	0.40	1.14 (1.02,1.28)	0.02
≥ 180 min	1.05 (0.78,1.40)	0.76	1.13 (0.99,1.28)	0.06
*Surgeon characteristics*				
Female gender (Ref: Male)	1.23 (1.04,1.45)	0.02	1.17 (1.08,1.26)	< 0.001
Patient volume (Ref: Low)				
Medium	0.71 (0.58,0.88)	0.001	0.93 (0.84,1.04)	0.22
High	0.74 (0.61,0.90)	0.003	0.83 (0.75,0.92)	< 0.001
No. hospital affiliations^b^ (Ref: 1)				
2	1.15 (0.97,1.36)	0.10	1.06 (0.99,1.14)	0.11
3 or more	1.29 (1.01,1.64)	0.04	0.99 (0.89,1.11)	0.89
*Regional characteristics*				
Region (Ref: South)				
Midwest	0.82 (0.68,0.98)	0.03	0.87 (0.78,0.98)	0.02
Northeast	1.20 (0.95,1.53)	0.12	1.25 (1.11,1.40)	< 0.001
West	1.16 (0.92,1.46)	0.21	1.17 (1.03,1.32)	0.01
Area deprivation index (Ref: Quartile 1)				
Missing	1.31 (0.61,2.81)	0.48	1.27 (0.68,2.37)	0.45
Quartile 2	1.41 (0.99,2.00)	0.05	1.09 (1.00,1.18)	0.04
Quartile 3	1.30 (0.91,1.84)	0.14	1.08 (0.98,1.18)	0.12
Quartile 4	1.39 (0.97,1.99)	0.07	1.20 (1.08,1.34)	< 0.001
Surgery RUCA (Ref: Small rural town)				
Isolated small rural town	1.01 (0.56,1.79)	0.99	1.71 (0.60,4.85)	0.31
Large rural city/town	0.91 (0.68,1.22)	0.52	1.59 (0.73,3.47)	0.24
Urban	1.00 (0.74,1.36)	0.98	1.90 (0.92,3.92)	0.08

Mixed-effects logistic regression of surgical delay greater 90 days. Random effects for surgeon and patient county of residence

*CI* Confidence interval, *OR* Odds ratio, *NCI* National Cancer Institute, *NH* Non-hispanic, *RUCA* Rural-urban commuting area

^a^“Other” aggregates patients whose race/ethnicity is identified by the Research Triangle Institute algorithm as: American Indian/Alaskan Native, Other, or Unknown

^b^Number of unique hospitals at which a surgeon operated on an early-stage breast cancer patient irrespective of reconstruction status in the year of the patient’s surgery

Although excluded from the primary analysis, 10,209 patients underwent immediate reconstruction (Supplemental Table 4). The median biopsy-to-surgery interval was 39 days (IQR 27–54), with 17.8% experiencing intervals > 60 days and 4.4% having delays > 90 days. Compared with those receiving surgical treatment without immediate reconstruction, these patients were younger (median age 71, IQR 68–75 vs. 75, IQR 70–80), more frequently lived in urban areas (87.6% vs. 80.6%) and areas with lower social deprivation (median 31, IQR 14–56 vs. 43, IQR 22–67), and almost exclusively were treated in urban areas (96.2% vs. 88.5%).

Associations with delay among patients undergoing immediate reconstruction differed from those seen in nonreconstruction patients (Supplemental Table 4). As compared to NH-white patients, nonwhite patients had substantially increased risks of > 60-day and > 90-day delays, with NH-black patients having nearly three times the odds of NH-white patients of > 90-day delays (OR = 2.61, 95% CI = 1.91–3.57). We found no association between travel time and delayed surgical treatment that included immediate reconstruction (Supplemental Table 5). Stratifying for rurality (Supplemental Table 6), we found a high risk of delay > 90 days for NH-black rural patients undergoing immediate breast reconstruction (OR = 6.18, 95% CI = 0.94–40.8). Risk of delay with immediate reconstruction was increased among non-white urban-residing patients. Urban-residing patients with two or more additional clinical encounters prior to surgery had considerably increased chances of > 90-day delays (OR = 4.21, 95% CI = 3.21–5.51), although the effect was less pronounced than that among rural patients (OR = 7.81, 95% CI = 2.80–21.80). Whereas 36.1% of non-reconstruction patients were operated on by a female surgeon, 44.3% of reconstruction patients had female surgeons and no association between delay and surgeon gender was observed among these patients. Being operated on by a high-volume surgeon was only protective against delay among urban reconstruction patients (OR = 0.61, 95% CI = 0.44–0.84). No effect was seen for reconstruction surgeons with multiple hospital affiliations.

## Discussion

In this analysis of delays from biopsy to initial breast cancer surgical treatment, we found 1.9% of rural patients and 2.5% of urban patients experienced delays > 90 days and patient, surgeon, hospital, and regional factors associated with delays varied in these two populations.

Our findings are consistent with recent work showing differences in delays and surgical procedure types among rural and urban breast cancer patients and resemble previously reported patterns of delays by procedure and region.^18,22^ Factors common across patient strata included increased odds of delay for patients of NH-black or Hispanic race/ethnicity, older patients, patients with additional comorbidities, and those having additional clinic visits before surgery. NH-black and Hispanic women are more frequently diagnosed at advanced stages or with molecular subtypes, such as triple-negative breast cancers, that may necessitate additional consults, transfers of care, and more extensive surgery.^34^ Yet the increased risks of delay among NH-black and Hispanic women after adjusting for number preoperative clinic visits, as well as increased likelihood of delay for urban patients in the highest quartile of ADI, may be indicators of extant racial and socioeconomic disparities in access to care. Delays among older and/or sicker patients may be explained by care needed for other conditions or additional workup in anticipation of undergoing general anesthesia. Our observation of longer delays for patients treated at a teaching hospital or NCI cancer center, both of which attract more complex cases, may be related to transfers of care, also known to increase surgical delay.^10^ These effects persisted even when controlling for additional clinical encounters.

Rural patients typically live farther from cancer care services, raising questions as to whether greater travel time among rural patients would lead to delays in care; however, this was not observed in our study.^35,36^ We observed a 13–14% increase in the odds of delay for urban non-reconstruction patients with driving times ≥ 60 minutes, which may have confounded associations between facility distance and delay in prior studies that did not stratify by patient rurality. Delays did appear to be more likely to occur among rural patients having additional physician encounters before surgery compared with their urban counterparts, however, which may be consistent with greater challenges in access to specialty care or providers offering second opinions. Additional research, including more detailed information on patient travel and care sequence patterns, is needed to understand the association between driving time and surgical delay among urban patients. Although some studies have suggested rural breast cancer patients may be more likely than urban patients to be diagnosed with advanced disease within certain regions of the United States, several nationwide cohort studies did not detect such an association.^37^ Furthermore, differences in stage and grade at diagnosis appeared in a prior analysis of Medicare beneficiaries with nonmetastatic invasive breast cancer to explain fewer than 10 days of the variation in preoperative delay—and increases in both measures were associated with reductions in time-to-surgery.^19^ Surgical delay trends across histopathologic characteristics and clinical stages in that cohort and patient samples similar to ours suggest differences in stage are unlikely to explain our finding that > 90-day delays occurred more than 30% as often for urban nonreconstruction patients than among their rural counterparts.^12–14,19^

Odds of delay also varied by surgeon characteristics. In our analysis, rural patients of surgeons performing breast cancer-directed surgery at three or more hospitals had a 29% higher chance of delay than patients of surgeons only observed to operate at one hospital. Surgeons with multiple affiliations may increase access to care by providing specialized services to area hospitals, including those with rural catchment areas; yet our results suggest that in some cases this may come at the expense of timely care. Such surgeons also may provide locum tenens coverage to smaller rural hospitals, although the practice is difficult to capture in claims.^38^ Rural breast cancer patients are less likely to be treated by high-volume surgeons;^32,39^ yet our results demonstrate that those who do are significantly more likely to receive timely care. Evidence indicates that high-volume breast surgeons provide higher-quality care not explained by facility volume alone,^40^ although the relationship between surgeon volume and outcomes is complex.^32,41^ A recent study by Nattinger et al. found New York State’s 2009 policy limiting Medicaid reimbursements to facilities where ≥ 30 all-payer breast cancer operations were performed annually appeared to improve survival for both Medicaid and Medicare patients.^42^ If future regionalization efforts supporting dedicated breast cancer programs also consider surgeon caseloads, benefits to rural patients may be optimized.

Lastly, patients with female breast surgeons tended to experience longer delays. Female breast surgeons are more likely to have higher case volumes and may vary from men in subspecialty or the type of procedures they tend to perform, such as breast reconstruction.^39,41^ A recent survey of surgeons at academic and tertiary-care hospitals also indicated that female surgeons were less likely to receive operating room scheduling blocks sufficient for their caseloads.^43^ Patient preferences for same-gender physicians also may play a role in physician availability and patient decision-making.^44^

Our study has several limitations. Breast cancer studies of the U.S. fee-for-service Medicare population are not representative of all breast cancer cases in the United States and are limited by age requirements, lower representation of minority populations, and variations in Medicare Advantage participation. Prior work in the Medicare population found the likelihood of a male breast cancer patient experiencing a delay was at or below the level observed among women.^13,19^ Because the claims-based algorithms that we used have only been validated using female patient cohorts, our analysis was limited to female breast cancer patients and our findings may not extend to men being treated for breast cancer. Furthermore, staging, histopathologic grading, tumor size, molecular subtype, and genomic testing results, unavailable in claims data, have predictive value for modeling time to treatment and selection of early-stage patients most likely to experience worsened survival with delay.^11–15^ To address this, we excluded patients with claims that might reflect distant metastases, a method that despite having modest sensitivity (72.8%) has a greater than 94% negative predictive value.^45^ Our analysis did not differentiate between types of additional encounters during the biopsy-to-surgery interval, such as imaging, diagnostic work-ups, or transfers of care, which are known to contribute to delays.^8,19^ We also were unable to account for patients who did not receive care due to distance or socioeconomic hardship, which may have differentially affected rural and urban breast cancer patients. Geographic limitations of our study included our use of aggregation to ZIP codes and ZCTAs to obtain estimates of ADI, rurality, and travel time. Finally, by dichotomizing rural-urban status, we may have obscured differences at the far end of the rural spectrum.^46^

## Conclusions

Worsened survival among breast cancer patients has been associated with biopsy-to-surgery intervals greater than 90 days in patients across a range of clinical stages and phenotypes. Negative effects of surgical delay on survival are observed in both younger^47^ and older breast cancer patients.^4,12–14^ Despite delays increasing in recent years,^18,19,21^ interventions to address this issue have remained limited in scope.^48–50^ Although the necessities of a patient’s diagnostic work-up, decision-making regarding surgical preferences, and logistics required for scheduling procedures of different types are sometimes appropriate contributors to surgical delays in early breast cancer care, disparities in the timeliness of care exist that may warrant further consideration of quality measures or clinical guidelines, to protect at-risk patient populations. Future investigations and interventions should consider how drivers of delay may vary along the rural-urban continuum and how breast surgeon staffing and scheduling may be managed to more equitably facilitate timely care. We concur with the proposals of Obeng-Gyasi and colleagues in their recommendation that studies on the impact of COVID-19-related surgical delays and guideline changes should examine vulnerable populations independently by race and ethnicity, accounting for social determinants of health, treatment choices, and patient and institution-related reasons for delays.^51^ We encourage the use of stratification to discern which factors affect which populations most. The recent introduction of risk stratification approaches designed to help prioritize surgical scheduling for breast cancer patients with the highest risk of progression or other avoidable sequelae may represent an important step toward minimizing adverse effects associated with delays.^50^

## Supplementary Information

Below is the link to the electronic supplementary material.

Supplementary file1 (PDF 249 KB)

## Single-Sex Study Justification Statement

This study employs a claims-based, registry-validated approach to identifying incident breast cancer diagnoses among female Medicare beneficiaries. Given its unknown sensitivity and specificity among male beneficiaries, and that prior research on surgical delays among newly diagnosed breast cancer patients has focused on women patients, we similarly focused our analysis on female patients.
