# Supplementary material for: Rural-Urban Differences in Breast Cancer Surgical Delays in Medicare Beneficiaries
Source: Ann Surg Oncol. 2022 May 24;29(9):5759–69. doi: 10.1245/s10434-022-11834-4 (PMC9128633; doi:10.1245/s10434-022-11834-4)
Supplement: Supplementary file 1 — Supplementary file1 (PDF 249 KB) [file 10434_2022_11834_MOESM1_ESM.pdf]

Supplemental Appendix 1. Driving Time Estimation

Because ZIP codes changed during the study period, some OD pairs were unavailable in the matrix, and we assigned these ZIP codes to their nearest ZCTA. Finally, using latitudes and longitudes of the geometric centroids of the origin ZCTA (a, b) and destination ZCTA (c, d)[1], we interpolated driving times for remaining OD pairs not in the matrix by (1) calculating geodesic distances using the following equation[2]:

$$d_{OZCTA,DZCTA} = r\cos^{-1}[\sin\left(\frac{\pi a}{180}\right)\sin\left(\frac{\pi c}{180}\right) + \cos\left(\frac{\pi a}{180}\right)\cos\left(\frac{\pi c}{180}\right)\cos\left(\frac{\pi d}{180} - \frac{\pi b}{180}\right)] \quad (1)$$

where geographic coordinates were in decimal degrees and r represented an average Earth radius of 6367.4 km, then (2) estimated OD travel times in minutes ($t_{OZCTA,DCTA}$) using an empirically-derived regression model[3]:

$$t_{OZCTA,DCTA} = 33.91 + 0.66\ d_{OZCTA,DZCTA} \quad (2)$$

References

1. US Census Bureau. Gazetteer Files - ZIP Code Tabulation Areas. https://www.census.gov/geographies/reference-files/time-series/geo/gazetteer-files.html. Accessed June 18, 2021.

2. Wang F. *Quantitative Methods and Socio-Economic Applications in GIS*. Second edition. Boca Raton, FL: CRC Press; 2015.

3. Hu Y, Wang C, Li R, Wang F. Estimating a large drive time matrix between ZIP codes in the United States: A differential sampling approach. *Journal of Transport Geography*. 2020;86:102770. doi:10.1016/j.jtrangeo.2020.102770

Supplemental Table 1. Claims Codes Used to Identify Incident Breast Cancer Diagnoses

Diagnosis	**ICD9 diagnosis**	**174.xx, 233.0**
Biopsy	ICD9 procedure	8511
	HCPCS CPT	10021, 10022, 19081, 19082, 19083, 19084, 19085, 19086, 19100, 19101, 19102, 19103, 19110, 38792, 76095, 76942, 77012, 77031, 77032, 88170, 88305, 88309
Surgery	ICD9 procedure	8520, 8521, 8522, 8523, 8524, 8525, 8533, 8534, 8535, 8536, 8541, 8542, 8543, 8544, 8545, 8546, 8547, 8548
	HCPCS CPT	19112, 19120, 19125, 19126, 19160, 19162, 19180, 19182, 19200, 19220, 19240, 19260, 19271, 19272, 19301, 19302, 19303, 19304, 19305, 19306, 19307, 38500, 38505, 38525, 38740, 38745, 38792

Abbreviations: CPT, Common Procedural Terminology; HCPCS, Healthcare Common Procedure Coding System; ICD9, *International Classification of Diseases, 9th Revision*

Supplemental Table 2. Claims Codes for Chemotherapy, Metastatic Disease, Needle Biopsies, and Definitive Surgical Treatment

	ICD9 diagnosis	ICD9 procedure	HCPCS CPT	RCC
Chemotherapy		V581, 9925	36640, 51720, 96400, 96405, 96405, 96406, 96408, 96410, 96412, 96414, 96420, 96422, 96423, 96425, 96440, 96445, 96450, 96501, 96504, 96505, 96508, 96520, 96524, 96530, 96538, 96540, 96542, 96545, 96549, 96555, 96510–96512, Q0083–Q0085, J9000–J9999	0331, 0332, 0335
Metastatic Disease	1962, 1965, 1966, 197, 1970, 1971, 1972, 1973, 1974, 1975, 1976, 1977, 1978, 198, 1980, 1981, 1982, 1983, 1984, 1985, 1986, 1987, 1988, 19881, 19882, 19889			
Needle biopsy		8511	10021, 10022, 19081, 19082, 19083, 19084, 19085, 19086, 19100, 19102, 19103	
Lumpectomy		8520, 8521, 8522, 8523, 8524, 8525	19120, 19125, 19126, 19160, 19301	
Lumpectomy with lymphadenectomy			19162,19302	
Mastectomy*		8533, 8534, 8535, 8536, 8541, 8542	19180, 19182, 19303, 19304	
Mastectomy and lymphadenectomy		8543, 8544, 8545, 8546, 8547, 8548	19200, 19220, 19240, 19305, 19306, 19307	
Lymph node resection/lymphadenectomy†		4011, 4022, 4023, 4029, 403, 4050, 4051	38500, 38525, 38530, 38740, 38745	
Sentinel lymphadenectomy†		4019, 8519, 9216	38790, 38792, 38900, 78195	

Abbreviations: CPT, Common Procedural Terminology; HCPCS, Healthcare Common Procedure Coding System; ICD9, *International Classification of Diseases, 9th Revision*; RCC, Revenue Center Codes

To be classified as definitive surgical treatment with therapeutic intent, mastectomies must include a lymph node procedure or, if coded individually (*), be accompanied by a lymph node procedure code (†).

Supplemental Table 3. Models of Non-Reconstruction Surgical Delays >60 Days[a]

N = 238,491	Crude OR (95% CI)	*P*	Adjusted OR (95% CI)	*P*
**Patient Characteristics**				
Age (Ref: 66-69)				
70-74	1.04 (0.99,1.09)	0.10	1.06 (1.01,1.12)	0.015
75-79	1.12 (1.06,1.17)	<0.001	1.19 (1.13,1.25)	<0.001
80-99	1.33 (1.26,1.39)	<0.001	1.54 (1.47,1.62)	<0.001
Race/Ethnicity (Ref: NH-white)				
Asian/Pacific Islander	1.07 (0.92,1.24)	0.40	1.08 (0.92,1.26)	0.33
Hispanic	1.83 (1.58,2.12)	<0.001	1.74 (1.49,2.02)	<0.001
NH-black	2.00 (1.89,2.11)	<0.001	1.88 (1.78,1.99)	<0.001
Other[b]	1.39 (1.23,1.56)	<0.001	1.40 (1.24,1.58)	<0.001
Charlson comorbidities (Ref: 0)				
1	1.26 (1.21,1.31)	<0.001	1.25 (1.20,1.30)	<0.001
2 or more	1.84 (1.77,1.92)	<0.001	1.85 (1.77,1.92)	<0.001
Preoperative clinician visits (Ref: 0)				
1	1.40 (1.34,1.46)	<0.001	1.53 (1.47,1.60)	<0.001
2 or more	3.70 (3.55,3.86)	<0.001	4.08 (3.91,4.26)	<0.001
Treated at teaching hospital	1.46 (1.38,1.55)	<0.001	1.22 (1.14,1.29)	<0.001
Treated at NCI cancer center	1.97 (1.80,2.16)	<0.001	1.50 (1.37,1.65)	<0.001
Estimated driving time to hospital (Ref: ≤29 min)				
30-59 min	1.02 (0.98,1.06)	0.31	1.04 (1.00,1.08)	0.048
60-179 min	1.14 (1.08,1.21)	<0.001	1.17 (1.10,1.24)	<0.001
≥180 min	1.24 (1.16,1.32)	<0.001	1.22 (1.14,1.30)	<0.001
**Surgeon Characteristics**				
Female gender (Ref: Male)	1.36 (1.29,1.44)	<0.001	1.22 (1.16,1.29)	<0.001
Patient volume (Ref: Low)				
Medium	0.89 (0.84,0.94)	<0.001	0.87 (0.81,0.92)	<0.001
High	0.97 (0.92,1.03)	0.31	0.84 (0.79,0.89)	<0.001
Number of hospital affiliations[c] (Ref: 1)				
2	1.03 (0.98,1.07)	0.26	1.05 (1.00,1.09)	0.05
3 or more	1.06 (0.99,1.13)	0.08	1.05 (0.98,1.12)	0.14
**Regional Characteristics**				
Region (Ref: South)				
Midwest	0.81 (0.75,0.88)	<0.001	0.91 (0.84,0.98)	0.01
Northeast	1.58 (1.45,1.72)	<0.001	1.54 (1.43,1.67)	<0.001
West	1.25 (1.15,1.37)	<0.001	1.35 (1.25,1.47)	<0.001
Area Deprivation Index (Ref: Quartile 1)				
Missing	1.13 (0.85,1.49)	0.39	1.10 (0.83,1.46)	0.50
Quartile 2	1.06 (1.01,1.11)	0.01	1.05 (1.00,1.10)	0.03
Quartile 3	1.10 (1.04,1.16)	0.001	1.09 (1.03,1.15)	0.001
Quartile 4	1.28 (1.21,1.36)	<0.001	1.19 (1.12,1.26)	<0.001
Patient RUCA (Ref: Small rural town)				
Isolated Small Rural Town	1.10 (0.97,1.25)	0.12	1.10 (0.97,1.24)	0.15
Large Rural City/Town	1.04 (0.93,1.16)	0.47	1.10 (0.98,1.22)	0.10
Urban	1.16 (1.05,1.27)	0.002	1.11 (1.01,1.22)	0.03
Surgery RUCA (Ref: Small rural town)				
Isolated Small Rural Town	1.16 (0.83,1.63)	0.39	0.96 (0.68,1.34)	0.79
Large Rural City/Town	1.01 (0.85,1.21)	0.89	0.96 (0.80,1.16)	0.68
Urban	1.44 (1.23,1.69)	<0.001	1.15 (0.98,1.36)	0.09

Abbreviations: CI, confidence interval; NCI, National Cancer Institute; NH, non-Hispanic; OR, odds ratio; RUCA, rural-urban commuting area.

[a] Mixed-effects logistic regression of surgical delay greater than 60 days or 90 days. Random effects for surgeon and patient county of residence.
[b] "Other" aggregates patients whose race/ethnicity is identified by the Research Triangle Institute algorithm as: American Indian/Alaskan Native, Other, or Unknown.
[c] The number of unique hospitals at which a surgeon operated on an early-stage breast cancer patient irrespective of reconstruction status in the year of the patient's surgery.

Supplemental Table 4. Patient Characteristics by Surgical Delay Status Among Patients Undergoing Immediate Breast Reconstruction

	≤60 Days	>60 & ≤90 Days	>90 & ≤180 Days	*P*[a]
N	8391	1365	453	
**Age (%)**				<0.001
66-69	3287 (39.2)	552 (40.4)	190 (41.9)	
70-74	2894 (34.5)	516 (37.8)	145 (32.0)	
75-79	1403 (16.7)	216 (15.8)	71 (15.7)	
80-99	807 (9.6)	81 (5.9)	47 (10.4)	
**Race/Ethnicity (%)**				<0.001
NH-black	425 (5.1)	135 (9.9)	68 (15.0)	
NH-white	7754 (92.4)	1177 (86.2)	359 (79.2)	
Other[b]	212 (2.5)	53 (3.9)	26 (5.7)	
**Charlson comorbidities (%)**				0.05
0	5439 (64.8)	851 (62.3)	275 (60.7)	
1	2047 (24.4)	345 (25.3)	113 (24.9)	
2 or more	905 (10.8)	169 (12.4)	65 (14.3)	
**Preoperative clinician visits (median [IQR])**	1 [0,1]	1 [0,3]	2 [1,3]	<0.001
**Teaching hospital (%)**	2000 (23.8)	432 (31.6)	146 (32.2)	<0.001
**NCI cancer center (%)**	709 (8.4)	183 (13.4)	74 (16.3)	<0.001
**Est. driving time to hospital**				0.12
≤29 min	2898 (34.5)	435 (31.9)	172 (38.0)	
30-59 min	3297 (39.3)	549 (40.2)	158 (34.9)	
60-179 min	1304 (15.5)	236 (17.3)	68 (15.0)	
≥180 min	892 (10.6)	145 (10.6)	55 (12.1)	
**Region (%)**				<0.001
Midwest	1632 (19.4)	244 (17.9)	51 (11.3)	
Northeast	1570 (18.7)	302 (22.1)	105 (23.2)	
South	3475 (41.4)	522 (38.2)	179 (39.5)	
West	1714 (20.4)	297 (21.8)	118 (26.0)	
**Patient RUCA (%)**				0.01
Isolated Small Rural Town	227 (2.7)	35 (2.6)	.	
Small Rural Town	291 (3.5)	33 (2.4)	.	
Large Rural City/Town	557 (6.6)	86 (6.3)	16 (3.5)	
Urban	7316 (87.2)	1211 (88.7)	420 (92.7)	

Abbreviations: IQR, interquartile range; NCI, National Cancer Institute; NH, non-Hispanic; RUCA, rural-urban commuting area.

. = Suppressed due to counts below 11
[a] Chi-squared test *p*-values for categorical variables, Kruskal-Wallis test *p*-values for medians
[b] "Other" aggregates patients whose race/ethnicity is identified by the Research Triangle Institute algorithm as: American Indian/Alaskan Native, Asian/Pacific Islander, Hispanic, Other, or Unknown.

Supplemental Table 5. Models of Surgical Delay for Patients Undergoing Immediate Breast Reconstruction[a]

	>60-Day Delay				>90-Day Delay			
N = 10,209	**Crude OR (95% CI)**	***P***	**Adjusted OR (95% CI)**	***P***	**Crude OR (95% CI)**	***P***	**Adjusted OR (95% CI)**	***P***
**Patient Characteristics**								
Age (Ref: 66-69)								
70-74	1.03 (0.91,1.17)	0.66	1.05 (0.92,1.20)	0.44	0.87 (0.69,1.10)	0.23	0.89 (0.70,1.12)	0.31
75-79	0.95 (0.81,1.12)	0.53	1.03 (0.87,1.22)	0.76	0.90 (0.68,1.21)	0.49	0.99 (0.73,1.33)	0.93
80-99	0.74 (0.59,0.92)	0.006	0.89 (0.71,1.12)	0.32	1.11 (0.78,1.57)	0.56	1.39 (0.97,2.00)	0.07
Race/Ethnicity (Ref: NH-white)								
NH-black	2.16 (1.75,2.65)	<0.001	2.08 (1.68,2.58)	<0.001	2.94 (2.18,3.97)	<0.001	2.61 (1.91,3.57)	<0.001
Other[b]	1.80 (1.34,2.41)	<0.001	1.64 (1.21,2.22)	0.001	2.33 (1.50,3.62)	<0.001	1.94 (1.23,3.05)	0.004
Charlson comorbidities (Ref: 0)								
1	1.09 (0.96,1.24)	0.19	1.13 (0.99,1.29)	0.07	1.08 (0.86,1.36)	0.51	1.09 (0.86,1.39)	0.47
2 or more	1.25 (1.05,1.49)	0.009	1.39 (1.16,1.66)	<0.001	1.38 (1.03,1.84)	0.03	1.36 (1.00,1.85)	0.05
Preoperative clinician visits (Ref: 0)								
1	1.41 (1.21,1.64)	<0.001	1.41 (1.21,1.64)	<0.001	1.28 (0.95,1.74)	0.10	1.32 (0.97,1.79)	0.07
2 or more	3.99 (3.47,4.58)	<0.001	3.84 (3.33,4.43)	<0.001	4.48 (3.48,5.77)	<0.001	4.40 (3.39,5.71)	<0.001
Treated at teaching hospital	1.43 (1.23,1.65)	<0.001	1.20 (1.02,1.40)	0.02	1.45 (1.15,1.83)	0.001	1.23 (0.95,1.59)	0.11
Treated at NCI cancer center	1.75 (1.43,2.15)	<0.001	1.25 (0.99,1.56)	0.05	1.94 (1.43,2.63)	<0.001	1.43 (1.02,1.99)	0.04
Estimated driving time to hospital (Ref: ≤29 min)								
30-59 min	1.07 (0.93,1.22)	0.35	1.09 (0.95,1.25)	0.23	0.81 (0.64,1.02)	0.07	0.87 (0.69,1.11)	0.24
60-179 min	1.15 (0.97,1.37)	0.11	1.18 (0.99,1.42)	0.06	0.86 (0.64,1.17)	0.33	0.93 (0.68,1.27)	0.62
≥180 min	1.12 (0.92,1.37)	0.26	1.08 (0.88,1.33)	0.45	1.02 (0.73,1.42)	0.92	1.02 (0.72,1.43)	0.93
**Surgeon Characteristics**								
Female gender (Ref: Male)	1.31 (1.15,1.50)	<0.001	1.09 (0.95,1.26)	0.22	1.32 (1.06,1.63)	0.01	1.18 (0.94,1.48)	0.16
Patient volume (Ref: Low)								
Medium	0.84 (0.66,1.05)	0.12	0.87 (0.69,1.10)	0.23	0.73 (0.50,1.06)	0.09	0.73 (0.50,1.08)	0.11
High	1.08 (0.90,1.30)	0.39	0.98 (0.80,1.19)	0.80	0.74 (0.55,0.99)	0.04	0.63 (0.45,0.86)	0.003
Number of hospital affiliations[c] (Ref: 1)								
2	0.97 (0.85,1.12)	0.70	1.00 (0.87,1.16)	0.97	0.96 (0.76,1.22)	0.76	1.05 (0.82,1.35)	0.69
3 or more	1.06 (0.88,1.27)	0.55	1.05 (0.87,1.27)	0.62	0.89 (0.65,1.23)	0.48	1.01 (0.73,1.40)	0.93
**Regional Characteristics**								
Region (Ref: South)								
Midwest	0.86 (0.70,1.05)	0.12	0.94 (0.76,1.15)	0.53	0.63 (0.45,0.89)	0.008	0.69 (0.49,0.98)	0.04
Northeast	1.50 (1.24,1.83)	<0.001	1.28 (1.05,1.57)	0.01	1.29 (0.97,1.70)	0.07	1.06 (0.79,1.42)	0.68
West	1.41 (1.15,1.72)	0.001	1.41 (1.15,1.73)	0.001	1.44 (1.10,1.89)	0.008	1.34 (1.01,1.77)	0.04

Abbreviations: CI, confidence interval; NCI, National Cancer Institute; NH, non-Hispanic; OR, odds ratio; RUCA, rural-urban commuting area.

[a] Mixed-effects logistic regression of surgical delay greater than 60 days or 90 days. Random effects for surgeon and patient county of residence.
[b] "Other" aggregates patients whose race/ethnicity is identified by the Research Triangle Institute algorithm as: American Indian/Alaskan Native, Asian/Pacific Islander, Hispanic, Other, or Unknown.
[c] The number of unique hospitals at which a surgeon operated on an early-stage breast cancer patient irrespective of reconstruction status in the year of the patient's surgery.

Supplemental Table 6. Model of Over 90-Day Surgical Delay for Patients Undergoing Immediate Breast Reconstruction, Stratified by Rurality[a]

	Rural (N = 1262)		Urban (N = 8947)	
	**Adjusted OR (95% CI)**	***P***	**Adjusted OR (95% CI)**	***P***
**Patient Characteristics**				
Age (Ref: 66-69)				
70-74	0.85 (0.33,2.22)	0.74	0.88 (0.69,1.13)	0.31
75-79	0.58 (0.14,2.42)	0.45	1.00 (0.73,1.35)	0.97
80-99	3.21 (0.88,11.74)	0.07	1.26 (0.86,1.86)	0.22
Race/Ethnicity (Ref: NH-white)				
NH-black	6.18 (0.94,40.81)	0.05	2.53 (1.84,3.48)	<0.001
Other[b]	2.97 (0.41,21.34)	0.27	1.92 (1.21,3.06)	0.005
Charlson Comorbidities (Ref: 0)				
1	1.38 (0.55,3.50)	0.49	1.08 (0.84,1.38)	0.55
2 or more	2.00 (0.58,6.98)	0.27	1.33 (0.96,1.82)	0.08
Preoperative clinician visits (Ref: 0)				
1	1.46 (0.41,5.16)	0.55	1.29 (0.94,1.77)	0.11
2 or more	7.81 (2.80,21.77)	<0.001	4.21 (3.21,5.51)	<0.001
Treated at teaching hospital	1.42 (0.51,4.01)	0.50	1.21 (0.93,1.57)	0.15
Treated at NCI cancer center	1.89 (0.53,6.72)	0.32	1.33 (0.94,1.87)	0.10
Est. driving time to hospital (Ref: ≤29 min)				
30-59 min	0.47 (0.08,2.72)	0.39	0.88 (0.69,1.12)	0.29
60-179 min	0.38 (0.08,1.82)	0.22	1.12 (0.80,1.58)	0.51
≥180 min	0.57 (0.11,2.97)	0.50	1.12 (0.76,1.63)	0.57
**Surgeon Characteristics**				
Female gender (Ref: Male)	1.96 (0.79,4.88)	0.14	1.14 (0.90,1.43)	0.28
Patient volume (Ref: Low)				
Medium	1.89 (0.37,9.66)	0.44	0.69 (0.46,1.04)	0.07
High	1.04 (0.23,4.73)	0.96	0.61 (0.44,0.84)	0.002
Number of hospital affiliations[c] (Ref: 1)				
2	1.37 (0.52,3.61)	0.52	1.03 (0.80,1.33)	0.80
3 or more	0.86 (0.22,3.41)	0.83	1.03 (0.74,1.44)	0.84
**Regional Characteristics**				
Region (Ref: South)				
Midwest	0.69 (0.24,1.97)	0.48	0.71 (0.49,1.03)	0.07
Northeast	1.26 (0.35,4.46)	0.72	1.05 (0.78,1.41)	0.74
West	0.62 (0.17,2.26)	0.46	1.38 (1.03,1.83)	0.03

Abbreviations: CI, confidence interval; NCI, National Cancer Institute; NH, non-Hispanic; OR, odds ratio; RUCA, rural-urban commuting area.

[a] Mixed-effects logistic regression of surgical delay greater than 90 days. Random effects for surgeon and patient county of residence.
[b] "Other" aggregates patients whose race/ethnicity is identified by the Research Triangle Institute algorithm as: American Indian/Alaskan Native, Asian/Pacific Islander, Hispanic, Other, or Unknown.
[c] The number of unique hospitals at which a surgeon operated on an early-stage breast cancer patient irrespective of reconstruction status in the year of the patient's surgery.
